# Leisure time satisfaction as a predictor of life satisfaction among teachers

**DOI:** 10.3389/fpsyg.2026.1817731

**Published:** 2026-06-03

**Authors:** Fuat Karadayi, Aysem İyikal Çelebi, Hülya Şenol

**Affiliations:** 1 Business Administration Department, Cyprus Health and Social Sciences University, Mersin, Türkiye; 2 Education Administration and Inspection Department, Cyprus Health and Social Sciences University, Mersin, Türkiye

**Keywords:** life satisfaction, leisure satisfaction, teachers, private sector, pre-school, primary school, secondary school, high school

## Abstract

**Introduction:**

The aim of this quantitative study is to examine the relationship between life satisfaction and leisure time satisfaction among teachers working in the private education sector.

**Methods:**

The study's population includes 1,535 teachers employed at 115 private schools in the Turkish Republic of Northern Cyprus. A sample of 308 teachers was selected using a stratified sampling method. Data were collected through a personal information form, the Leisure Time Satisfaction Scale (LTSS), and the Life Satisfaction Scale (LSS). The data analysis was conducted using SPSS 26 software and parametric tests.

**Results and discussion:**

Female teachers, preschool educators, and full-time teachers in private schools tend to have higher satisfaction with their leisure activities. Furthermore, female teachers and those working full-time demonstrate significantly greater overall levels of life satisfaction. Correlation analysis indicates a strong and significant positive relationship between life satisfaction and leisure satisfaction. Furthermore, the regression analysis revealed a significant association between various sub-dimensions of leisure satisfaction and life satisfaction.

## Introduction

Life satisfaction is a complex concept that reflects how individuals assess their overall quality of life within psychological, social, and economic domains. It is influenced by various intrinsic and extrinsic factors, such as personal values, physical and mental health, financial stability, and social relationships (Öter and Dagli, 2022; Özavci et al., 2023). Recent research increasingly views life satisfaction as part of a broader framework of wellbeing, where both work-related and non-work-related experiences contribute to individuals' overall life evaluations. In workplace settings, particularly in professions like teaching, factors such as job demands, job resources, and organizational support are closely linked to wellbeing outcomes.

Teachers' wellbeing is part of a complex network that includes factors such as burnout, work engagement, occupational commitment, and turnover intentions. High workloads, emotional labor, and institutional expectations can put teachers at risk for stress and decrease their overall wellbeing. In this context, resources that come from outside the workplace become especially important. Leisure is one such resource; however, recent research indicates that it is not just the act of participating in leisure activities that matters, but rather how individuals evaluate these experiences—known as leisure satisfaction—that is more strongly linked to positive wellbeing outcomes.

Leisure satisfaction refers to the level of enjoyment and fulfillment that individuals experience from their leisure activities. It includes various dimensions such as psychological wellbeing, social interaction, education, and relaxation (Öztürk and Alpullu, 2023). Theoretically, leisure satisfaction can serve as a personal resource that contributes to stress recovery, emotional regulation, and social connectedness. Participating in meaningful leisure activities has been linked to lower stress levels, improved emotional wellbeing, and a better overall quality of life (Hjalmarsson and Löfdahl Hultman, 2015; Sevinç and Özel, 2018). However, the relationship between leisure and wellbeing is not always straightforward or consistent. Research indicates that the type of leisure activity, the context in which it occurs, and how individuals subjectively evaluate their experiences can all influence how these activities relate to broader wellbeing outcomes.

Leisure satisfaction may be linked to overall life satisfaction by enhancing individuals' psychological resources and influencing their evaluations of life. However, this relationship can vary based on both contextual and individual factors. For instance, employment conditions like job security and workload can affect not only access to leisure activities but also how satisfying those activities are. Similarly, a person's level of education may reflect different professional demands, while demographic factors such as gender, age, and educational background can contribute to variations in leisure preferences and overall wellbeing.

Although previous research has shown associations between leisure experiences and life satisfaction across various populations, there is limited research specifically focusing on teachers, particularly those in the private education sector. Additionally, the relationship between leisure satisfaction and life satisfaction within the broader context of teacher wellbeing, and how this relationship may vary based on socio-demographic and professional factors, has not been thoroughly explored.

Therefore, this study aims to investigate the relationship between leisure satisfaction and life satisfaction among private school teachers. It will also examine whether these relationships differ based on selected demographic and professional characteristics. By situating this analysis within the broader literature on teacher wellbeing, this study seeks to enhance our understanding of how leisure experiences relate to overall life evaluations in this professional group.

## Purpose of the research

The purpose of this study is to determine the relationship between life satisfaction and leisure-time satisfaction among teachers working in private educational institutions. In addition, answers to the following sub-questions were sought:

1. Do the leisure time satisfaction and life satisfaction levels of teachers working in the private sector differ significantly according to their socio-demographic characteristics?
2. What is the relationship between the leisure time satisfaction levels of private sector teachers and their overall life satisfaction?

## Importance of research

There has been extensive study on the relationship between life satisfaction and leisure satisfaction across many demographic groups; however, there is a lack of studies focusing on instructors within the private school sector. The existing literature is largely shaped by research addressing the associations between these two constructs and a range of socio-demographic and psychological variables in different sectors. For instance, research in which university students participated has examined the correlation between leisure satisfaction, life satisfaction, and the reasons for social networking use (Himmetoglu and Ayhan, 2021). Sports-related research has examined the connection between tennis players' life satisfaction and leisure management (Duran et al., 2020) and the impact of leisure limitations on life satisfaction (Çetinkaya and Akova, 2020), illustrating the connection between leisure management techniques and individual happiness. Studies examining emotional intelligence, leisure satisfaction, and life satisfaction further emphasize the importance of considering individual characteristics when evaluating leisure experiences (Yildirim and Latifoglu, 2020). Similarly, research investigating the association between participation in informational or skill-enhancing leisure activities, stress, and life satisfaction highlights the contribution of leisure to psychological wellbeing (Sabirli et al., 2022).

Research involving healthcare professionals has explored the relationship between quality of life, life satisfaction, and leisure satisfaction (Tokay Argan and Mersin, 2021). These studies suggest that specific dynamics within the sector influence leisure experiences. The literature also highlights the significance of leisure nostalgia in contributing to life satisfaction (Cho, 2020) and models that assess school life satisfaction through leisure activities among adolescents (Jung, 2020). Additionally, large-scale studies have examined the impact of leisure activities on subjective wellbeing (Mutz et al., 2021; Yoon et al., 2020; Nissen et al., 2025). Furthermore, research focusing on immigrants has shown that engaging in leisure activities can enhance life satisfaction and promote social integration (Berasategi Sancho et al., 2023), highlighting the social and cultural dimensions of leisure.

Although the relationship between leisure and life satisfaction has been extensively studied across various disciplines and populations, these concepts remain under-researched among private-sector teachers, particularly concerning the impact of their demographic profiles. The outcomes of this study aim to enhance comprehension of the variables influencing educators' quality of life and to offer significant insights for academic literature and decision-making processes. The study has the potential to improve policy formulation and institutional practices that enhance employee wellbeing in educational settings and to provide a foundation for future research.

## Theoretical framework

### Life satisfaction

Life satisfaction with a high level of life satisfaction refers to a multifaceted construct representing an individual's overall appraisal of her or his life according to various psychological, social, economic and cultural factors. As for teachers, their life satisfaction is not only affected by personal characteristics, such as age, sex, marital status and health conditions, but also by their occupational characteristics, social environment and leisure activities (Senyurt et al., 2023; Luque-Reca et al., 2022).

The factor which affects life satisfaction most is psychological health. Teachers experience various psychological characteristics including stress resistance, self-esteem and self-efficacy which predict life satisfaction (Marcionetti and Castelli, 2023). It is anticipated that the high level of psychological strength, self-esteem and self-efficacy among teachers will facilitate the dealing with stress and achieve the life satisfaction. High emotional competence and intrinsic job satisfaction were also expected to enhance life satisfaction of teachers (Luque-Reca et al., 2022).

Religious beliefs, cultural values and social support are also important psychosocial determinants of life satisfaction. Individuals who actively use religious coping mechanisms report higher levels of life satisfaction (Mercanlioglu, 2021). Life satisfaction was positively related to work performance and negatively related to organizational cynicism. These findings suggest that the life satisfaction–work–life balance relationship may be influenced by employees' organizational commitment and work performance. Life satisfaction proved to be a significant predictor of the variables in the proposed model, indicating that employees with higher levels of life satisfaction tend to be more involved in their work and less prone to displaying counterproductive work behavior. Recreational sports and life satisfaction besides work and family, many people value leisure activities highly when it comes to their life satisfaction. Positive relationship was found between recreational sports and life satisfaction (Mutz et al., 2021), between quality of life, life satisfaction and leisure satisfaction (Tokay Argan and Mersin, 2021), and between life satisfaction and leisure activities (Yoon et al., 2020). Besides contributing to the body's physical rehabilitation, social and leisure activities also support the individual's psychological recovery.

Life satisfaction is a vital construct both for individuals and for society. High life satisfaction is related to a number of beneficial effects, such as increased productivity, health and wellbeing and life satisfaction (Mensah et al., 2023). Examples of factors which may have a negative impact on life satisfaction are work–life conflict, stress and heavy workload, and which the individual can counteract with the use of psychological resources such as, for example, resilience and self-efficacy (Misfin et al., 2024). Finally we conclude that life satisfaction as a dynamic construct is mediated by psychological resilience, self-efficacy, emotional regulation, social support, leisure activities and work environment. It is also influenced by the meaning-making of religious and cultural values in order to protect individual wellbeing, organization performance and social wellbeing. Life satisfaction is very important for enhancing individual wellbeing and organization performance (Mercanlioglu, 2021).

## Leisure time satisfaction

Leisure satisfaction is a multi-dimensional trait which relates to the ways in which people utilize their leisure time and the extent to which they are satisfied with leisure (1). When individuals engage in their free time activities which provide them with satisfaction and enjoyment, this has a positive impact on life satisfaction and monotonous and dissatisfactory leisure activities tend to hinder the increase of life satisfaction. Therefore, leisure satisfaction activities are based on meeting individual needs, social interaction and self-improvement. Strengthening of the social relations and interpersonal communication can be beneficial for the psychological state and quality of life (Serdar et al., 2021) and satisfying the individual's needs and preferences have a positive impact on the level of life quality (Choi and Yoo, 2017).

Leisure satisfaction is another aspect of time effectiveness. While many people have to do some paid work in their leisure time, many others simply waste time doing nothing useful or are just inactive. TV and social media can grab our attention and be fun and fascinating at first, but are ultimately bad for our productivity and leisure satisfaction. Our time should be spent on more fun and leisure-satisfying activities, including more socialization and more learning, which in turn will bring us more leisure satisfaction (Serdar et al., 2021).

Physical activity and social interaction stand out as the most influential components of leisure satisfaction. A study conducted by Mutz et al. (2021) demonstrated that sports played in leisure time improves life satisfaction and subjective wellbeing. Another study revealed that the participation in physical activities for instance reformer Pilates mat work on a regular basis enhances leisure and life satisfaction (Karagöz, 2022). As a result, the physical activity has the benefits to our physical health and we experience emotional renewal and a reduction of stress.

Education research has found connections between the employees' leisure time happiness and work satisfaction. Research that examined the effect of teacher autonomy support on employees' leisure time physical activity showed that autonomy support in the workplace influences employees' cognitive evaluations and emotions related to physical activity, which in turn influences their leisure time physical activity (Zimmermann et al., 2021). In another study, it was found that teachers who participated in group-based leisure-time physical activity reported greater work happiness, motivation, engagement and job commitment (Abós et al., 2021). These studies are good examples of how leisure time activities increase the wellbeing of individuals and therefore also benefit the organizations they work for. That a leisure activity can affect teachers' knowledge and pedagogical performance in the school subjects they teach is perhaps not unexpected. On the other hand, that a leisure time activity can also affect the professional identity of a teacher as a leisure time teacher is perhaps more surprising. However, a large number of teachers believe that their leisure time can be very important for their identity, membership and work satisfaction as teachers.

Leisure satisfaction is determined by a wide range of variables, from physical environment and psychological characteristics to social and cultural context. Cultural differences in societal values and corresponding work–life balance policies lead to different leisure experiences and behavior (Andrade and Westover, 2023). While in some cultures, for example, spending time with the family or participating in communal activities is highly valued as leisure, in other cultures, for example, individual leisure activities or personal development are considered the main leisure sources. Consequently, leisure satisfaction cannot be treated as an individual phenomenon but is culturally constructed.

## Method

### Research model

This study utilized a relational (correlational) research design to examine the associations between teachers' leisure satisfaction and life satisfaction. Relational screening models are suitable for identifying the direction and strength of relationships among variables without implying causation (Karasar, 2020; Büyüköztürk et al., 2022). Thus, this study focused on the statistical associations between the main variables.

In addition to analyzing the overall relationship between leisure satisfaction and life satisfaction, subgroup analyses were conducted to explore whether these variables differed based on selected socio-demographic and professional characteristics, including gender, age, professional seniority, and school type. While this design allows for the identification of meaningful associations, it does not establish causal relationships. Instead, it provides a foundation for understanding how these variables are interrelated within the broader context of teachers' psychosocial wellbeing.

### Population and sample

The target population of this study included teachers working in private schools in the Turkish Republic of Northern Cyprus (TRNC). According to data from the Ministry of Education (2024), there are 115 private schools employing approximately 1,535 teachers across preschool, primary, secondary, and high school levels. Administrative and technical staff were not included in the study. A stratified sampling strategy was employed to ensure proportional representation across school levels (preschool, primary, middle, and high school) as well as key demographic characteristics. Based on a 95% confidence level and a ±5% margin of error, a minimum sample size of 308 teachers was determined to be sufficient for representing the population. Participants were recruited through school administrations, and invitations to participate in the study were distributed via institutional communication channels, such as email and messaging platforms. In total, 385 teachers were invited to participate. Of these, 312 returned the questionnaire. After data screening, 4 responses were excluded due to missing or incomplete information. The final analytical sample consisted of 308 teachers.

### Data collection tools

Data was collected from the participant teacher by using a survey. Survey consisted of three sections including personal information form, Leisure time satisfaction scale, and Life satisfaction scale.

### Personal information form

The personal information form included questions designed to gather data on the demographic characteristics of the participants. This encompassed information such as participants' gender, age, marital status, educational background, professional experience, the type of school they work in, the province or district of their institution, the length of time they have worked at their current institution, and the nature of their work.

### Leisure satisfaction scale (LTS)

The Leisure Satisfaction Scale (LSS) was developed by Beard and Ragheb (1980). The original version of the scale is the long form and the short form version was developed in 1992. This scale was adapted to Turkish by Gökçe and Orhan (2011) and the scale was introduced to the literature in Turkish under the name “Serve Time Satisfaction Scale”. The scale aims to determine the extent to which individuals get satisfaction from their free time activities and consists of a total of 24 items grouped into six sub-dimensions: Psychological dimension- items 1, 2, 3, 4; Educational dimension- items 5, 6, 7, and 8; Social dimension- items 9, 10, 11, and 12; Relaxation dimension- items 13, 14, 15, and 16; Physical dimension- items 17, 18, 19, and 20; and Aesthetic dimension- items 21, 22, 23, and 24. This is a 5-Likert type scale ((1) “almost never true”, (5) “almost always true”. Kaiser-Meyer-Olkin (KMO) test was applied to test the construct validity of the scale and the result was found to be 0.82. In addition, the general reliability coefficient of the scale (Cronbach Alpha) was calculated at the level of 0.97. These results show that the scale is a highly valid and reliable measurement tool.

### Life satisfaction scale (LSS)

Life satisfaction scale developed by Diener et al. (1985), is a one-dimensional, 7-point Likert-type scale consisting of 5 items, and its original reliability coefficient was found to be Cronbach Alpha 0.87 and its criterion-dependent validity was found to be 0.82. This scale was adapted to Turkish by Yetim (1993), the reliability of the scale was found to be 0.86. In addition, in another study conducted by Dal and Baysal (2016), the construct validity of the 5-point Likert-type life satisfaction scale was found to be 0.869 and the scale reliability was found to be 0.88. These findings show that the scale is a reliable and valid measurement tool.

The reliability analysis result of the scales in our study is below.

The reliability analysis shows that all scales have high internal consistency. The overall Leisure Satisfaction Scale demonstrates excellent reliability with a Cronbach's alpha of 0.90. Its sub-dimensions have reliability scores ranging from good to very good, with alpha values between 0.83 and 0.88. Similarly, the Life Satisfaction Scale also shows high reliability at 0.88. These results indicate that the measurement instruments used in this study are psycho metrically robust and appropriate for further analysis. CFA analysis was not conducted in the present data set because Leisure Satisfaction Scale and Life Satisfaction Scales have undergone CFA and other validation procedures by the authors who developed these scales (Table 1).

**Table 1:** Cronbach's alpha values of the scales.

Scale/sub-dimension	Number of items	Cronbach's α
Leisure satisfaction scale (general)	24	0.90
Psychological dimension	4	0.87
Educational dimension	4	0.85
Social dimension	4	0.86
Relaxation dimension	4	0.88
Physical dimension	4	0.84
Aesthetic dimension	4	0.83
Life satisfaction scale	5	0.88

### Data collection

The survey forms were delivered to the participants online via Google Forms, and via e-mail and face-to-face in 2025. Before the surveys were administered, first permission was received from the ethical committee of the researchers' university (Reference number KSTU/2025/015), second from the Ministry of Education. All participants were provided with an informed consent form based on voluntary participation and their approvals were obtained. The data collection process lasted approximately 5 weeks. During this process, first, the relevant educational institutions were contacted and the necessary official permissions were obtained, and then the surveys were delivered to teachers working at preschool, primary school, secondary school and high school levels. Data were collected anonymously and all participants' personal information was protected within the framework of confidentiality principles. Following a full explanation of the purpose of the study, informed consent was obtained from all participants. All were made aware of their right to withdraw from the study at any stage.

### Analysis of data

The data were analyzed using SPSS 26.0 (Statistical Package for the Social Sciences) software. First, normality analysis was performed. The Kolmogorov–Smirnov normality test was applied to evaluate the distribution of the data (Table 2).

**Table 2:** Kolmogorov–Smirnov normality test results.

Scale/subscale	K–S	*df*	*p*	Skewness	Kurtosis
LSS – psychological	0.031	308	0.000	0.17	0.58
LSS – educational	0.029	308	0.000	0.15	−0.15
LSS – social	0.037	308	0.000	0.11	−0.09
LSS – relaxation	0.031	308	0.000	−0.14	−0.14
LSS – physical	0.026	308	0.000	0.01	−0.17
LSS – total	0.034	308	0.000	0.09	0.04
Life satisfaction scale	0.042	308	0.000	−0.11	−0.02

*p* < 0.05.

The results of the Kolmogorov–Smirnov (K–S) test indicate that all variables significantly deviate from a normal distribution. For each subscale of the Leisure Satisfaction Scale (LSS) and the Life Satisfaction Scale, the *p*-values are reported as 0.000 (*p* < 0.001), which means we reject the null hypothesis of normality. Although the K-S statistics are relatively small (ranging from 0.026 to 0.042), the large sample size (*n* = 308) increases the test's sensitivity, leading to statistically significant results even for minor deviations from normality. The skewness values range from −0.14 to 0.17, indicating that the distributions are approximately symmetric. The kurtosis values range from −0.17 to 0.58, suggesting that there is no severe peakedness or flatness in the distributions. These values fall within commonly accepted thresholds (±1 or ±2), implying that despite the significant results from the K-S test, the distributions are approximately normal in practice (Table 2).

## Results

Demographic information of the participants.

The sample primarily consisted of women (65.9%), with a majority of married participants (60.1%). The largest age group represented was individuals aged 31 to 35 years (29.2%). Most participants held an undergraduate degree (47.1%), followed by those with a master's degree (34.7%). Regarding professional experience, the largest proportion had 6 to 10 years in their field (35.1%). Participants were fairly evenly distributed across different school types, though there was a slight concentration in primary schools (29.9%). Most had been employed at their current school for 1 to 3 years (39.9%), and the majority of participants were working full-time (70.1%; Table 3).

**Table 3:** Demographic information of teachers.

Variable	Category	*n*	%
Gender	Woman	203	65.9
	Male	105	34.1
Age	20–25	59	19.2
	26–30	63	20.5
	31–35	90	29.2
	36–40	57	18.5
	41+	39	12.7
Marital status	Single	123	39.9
	Married	185	60.1
Educational status	Undergraduate	145	47.1
	Master's	107	34.7
	Doctorate	31	10.1
	Other	25	8.1
Professional seniority	1–5 years	93	30.2
	6–10 years	108	35.1
	11–15 years	65	21.1
	16+ years	46	14.9
School type	Preschool	77	25.0
	Primary school	92	29.9
	Middle school	77	25.0
	High school	62	20.1
Working time (same school)	< 1 year	31	10.1
	1–3 years	123	39.9
	4–6 years	93	30.2
	7+ years	61	19.8
Way of working	Full time	216	70.1
	Part time	46	14.9
	Contractual	31	10.1
	Other	15	4.9

### Do the leisure time satisfaction levels of teachers working in the private sector differ significantly according to their sociodemographic characteristics?

In this research, mean Leisure Time Satisfaction Scale scores of the participant teachers were compared according to their socio-demographic features. Their mean scores according to their age, marital status, educational background, professional seniority, working duration in the same school didn't differ significantly. Only significant results were presented in this part.

Table 4 presents the results of the independent samples *t*-test conducted to determine whether there are significant differences in the Leisure Satisfaction Scale scores of the teachers according to their gender variable.

**Table 4:** Comparison of the sub-dimension and general scores of the leisure time satisfaction scale of teachers according to their gender (*t*-test results).

Scale	Gender	*n*	M	SD	*t*	*p*	Cohen's *d*
Psychological	Female	203	22.88	3.53	2.59	0.010	0.30
	Male	105	21.70	4.28			
Educational	Female	203	23.06	3.74	3.92	0.000	0.47
	Male	105	21.27	3.91			
Social	Female	203	22.97	3.73	0.97	0.333	0.10
	Male	105	22.57	4.10			
Relaxation	Female	203	23.12	3.90	2.85	0.005	0.36
	Male	105	21.69	4.21			
Physical	Female	203	22.44	3.65	1.14	0.256	0.12
	Male	105	21.99	4.15			
Aesthetic	Female	203	23.11	3.85	2.01	0.045	0.25
	Male	105	22.12	4.00			
Total	Female	203	137.58	14.23	3.10	0.002	0.37
	Male	105	132.16	15.11			

*p* < 0.05.

The results of the independent samples *t*-test revealed that female teachers reported significantly higher levels of leisure satisfaction than male teachers in several areas. Specifically, female teachers scored higher in the psychological (*p* = 0.010), educational (*p* < 0.001), relaxation (*p* = 0.005), and aesthetic (*p* = 0.045) subdimensions, as well as in overall leisure satisfaction (*p* = 0.002). However, no significant gender differences were observed in the social (*p* = 0.333) and physical (*p* = 0.256) subdimensions. However, the effect size values indicated that the magnitude of these differences was generally small. Cohen's *d* values ranged from 0.25 to 0.47 for the significant differences, with the largest effect observed in the educational subdimension. The effect sizes for the non-significant social and physical dimensions were very small. Therefore, although some statistically significant gender differences were observed, the practical magnitude of these differences appears to be limited (Table 4).

The results of the one-way ANOVA indicated that leisure satisfaction varied significantly by school type in two areas: the relaxation subdimension (*F* = 11.04, *p* = 0.002) and overall scores (*F* = 11.12, *p* = 0.003). In both instances, teachers in preschool and primary school settings reported higher scores compared to those in high school. *Post-hoc* comparisons showed that teachers working at the high school level had lower scores in the relaxation subdimension than teachers working at the preschool, primary school, and middle school levels. Similarly, in terms of overall leisure time satisfaction, high school teachers had lower scores compared to teachers working at the preschool, primary school, and middle school levels. However, no significant differences were observed among the school types for the psychological, educational, social, physical, or aesthetic subdimensions (*p* > 0.05). These findings suggest that while school type has a limited effect, it does significantly impact certain aspects of leisure satisfaction (Table 5).

**Table 5:** Comparison of the sub-dimension and general scores of the leisure time satisfaction scale of teachers according to their school type (ANOVA-test results).

Scale	School type	*n*	M	*F*	*p*	*n* ^2^	Difference
Psychological	Preschool	77	22.45	1.18	0.318	0.012	
	Primary school	92	22.98				
	Middle school	77	22.75				
	High school	62	22.22				
Educational	Preschool	77	22.71	0.94	0.423	0.009	
	Primary school	92	22.81				
	Middle school	77	22.90				
	High school	62	22.36				
Social	Preschool	77	22.55	1.07	0.360	0.010	
	Primary school	92	22.74				
	Middle school	77	22.72				
	High school	62	22.10				
Relaxation	Preschool	77	22.64	11.04	0.002	0.098	Pre-school > High school; Primary school > High school; Middle school > High school
	Primary school	92	22.88				
	Middle school	77	22.75				
	High school	62	22.31				
Physical	Preschool	77	22.40	0.89	0.446		
	Primary school	92	22.55				
	Middle school	77	22.64				
	High school	62	22.03				
Aesthetic	Preschool	77	22.61	0.85	0.470		
	Primary school	92	22.83				
	Middle school	77	22.72				
	High school	62	22.34				
Overall score	Preschool	77	135.38	11.12	0.003	0.099	Primary school > High school, middle school; Preschool > High school
	Primary school	92	136.68				
	Middle school	77	136.03				
	High school	62	134.01				

*p* < 0.05.

*Post-hoc* comparisons were conducted using Turkey's HSD test.

The ANOVA results reveal that teachers' leisure time satisfaction levels differed significantly according to working style in some sub dimensions and in the overall score. Significant differences by working style were found in the psychological, education, social, relaxation, physical sub dimensions and overall leisure time satisfaction score. In contrast, no significant difference by working style was found in the aesthetic dimension. When the effect sizes were examined, it is seen that the explanatory effect of working style on the sub dimensions of leisure time satisfaction and the overall score was low. Therefore, although statistically significant differences were found, the practical magnitude of these differences can be considered limited.

*Post-hoc* comparisons showed that part-time teachers had higher scores in psychological and relaxation sub-dimensions than other groups. For the overall leisure time satisfaction score, full time and part time teachers had higher scores than contractual teachers. However, it should be noted that although the ANOVA results were significant in some dimensions, clear group differences in the *post-hoc* comparisons remained limited. This supports the interpretation that differences related to working style generally had a weak effect (Table 6).

**Table 6:** Comparison of the sub-dimension and general scores of the leisure time satisfaction scale of teachers according to their working style (ANOVA-test results).

Scale	Way of working	n	M	F	p	n^2^	*Post-hoc* differences
Psychological	Full time	216	22.58	4.25	0.007	0.040	Part time > Contractual; Part time > Other
	Part time	46	22.95				
	Contractual	31	22.20				
	Other	15	21.80				
Educational	Full time	216	22.75	3.89	0.011	0.037	
	Part time	46	22.90				
	Contractual	31	22.30				
	Other	15	22.10				
Social	Full time	216	22.40	2.98	0.032	0.029	
	Part time	46	22.65				
	Contractual	31	22.00				
	Other	15	21.90				
Relaxation	Full time	216	22.70	5.12	0.003	0.048	Part time > Contractual; Part time > Other
	Part time	46	22.95				
	Contractual	31	22.10				
	Other	15	21.85				
Physical	Full time	216	22.55	3.70	0.014	0.035	
	Part time	46	22.70				
	Contractual	31	22.05				
	Other	15	21.90				
Aesthetic	Full time	216	22.80	2.50	0.060	0.024	
	Part time	46	22.85				
	Contractual	31	22.25				
	Other	15	22.10				
Overall score	Full time	216	135.75	4.05	0.008	0.038	Full time > Contractual; Part time > Contractual
	Part time	46	136.20				
	Contractual	31	133.80				
	Other	15	133.60				

*p* < 0.05.

### Do the Life satisfaction levels of teachers working in the private sector differ significantly according to their sociodemographic characteristics?

In Table 7, teachers' life satisfaction scores were analyzed according to demographic variables.

**Table 7:** Analysis of life satisfaction scale scores of teachers.

Variable	Group	*n*	M	Test	Statistic	*p*	Effect size	Significant difference
Gender	Female	203	24.50	*t*-test	2.45	0.015^*^	*d* = 0.29	Female > Male
	Male	105	23.20					
Age	20–25	59	24.10	ANOVA	1.88	0.115	*n*^2^ = 0.024	-
	26–30	63	23.90					
	31–35	90	23.85					
	36–40	57	23.70					
	41+	39	24.00					
Marital status	Single	123	23.80	*t*-test	1.02	0.309	*d* = 0.12	-
	Married	185	24.10					
Educational background	Undergraduate	145	23.70	ANOVA	3.55	0.015^*^	*n*^2^ = 0.034	PhD > Other
	Master's	107	24.10					
	Doctorate	31	25.00					
	Other	25	22.80					
Professional seniority	1–5 years	93	23.85	ANOVA	0.98	0.405	*n*^2^ = 0.010	-
	6–10 years	108	23.95					
	11–15 years	65	23.60					
	16+ years	46	24.20					
School type	Preschool	77	24.20	ANOVA	4.05	0.009^*^	*n*^2^ = 0.038	Preschool > High school
	Primary school	92	23.90					
	Middle school	77	23.80					
	High school	62	23.10					
Working time (same school)	< 1 year	31	23.70	ANOVA	1.12	0.344	*n*^2^ = 0.011	-
	1–3 years	123	24.00					
	4–6 years	93	23.85					
	7+ years	61	23.90					
Type of working	Full time	216	24.20	ANOVA	5.22	0.002^*^	*n*^2^ = 0.049	Full-time > Contract, Other
	Part time	46	23.90					
	Contractual	31	22.80					
	Other	15	22.50					

^*^p < 0.05 (statistically significant).

The results indicate that life satisfaction varies significantly based on gender, educational background, school type, and employment status (*p* < 0.05). However, no significant differences were observed concerning age, marital status, professional seniority, or tenure at the same school (*p* > 0.05). Specifically, female teachers reported higher life satisfaction than male teachers. Individuals with doctoral degrees scored higher in terms of life satisfaction compared to those with other educational qualifications. Additionally, preschool teachers had higher satisfaction scores than high school teachers, and full-time employees reported greater life satisfaction than those in contractual or other types of employment. The effect sizes for these variables were low, indicating that these variables had a limited role in explaining life satisfaction (Table 7).

### Is there a significant relationship between life satisfaction and leisure time satisfaction levels of teachers working in the private sector?

All variables exhibited positive and significant correlations (p < 0.01). Life satisfaction showed moderate to strong associations with all subdimensions of leisure satisfaction, with correlation coefficients ranging from *r* = 0.62 to 0.78. The strongest relationship was found for overall (general) leisure satisfaction. Furthermore, the leisure time scale subdimensions were highly intercorrelated, indicating a consistent and cohesive structure. These findings suggest that greater leisure satisfaction is linked to higher life satisfaction (Table 8).

**Table 8:** Correlations between life satisfaction and leisure satisfaction sub-dimensions and general scores (Pearson momentum correlation coefficient).

Variable	1	2	3	4	5	6	7	8
1. Life Satisfaction	1	0.72^**^	0.68^**^	0.75^**^	0.70^**^	0.65^**^	0.62^**^	0.78^**^
2. Psychological		1	0.81^**^	0.79^**^	0.77^**^	0.69^**^	0.66^**^	0.85^**^
3. Educational			1	0.74^**^	0.72^**^	0.68^**^	0.63^**^	0.83^**^
4. Social				1	0.75^**^	0.61^**^	0.60^**^	0.82^**^
5. Relaxation					1	0.65^**^	0.64^**^	0.83^**^
6. Physical						1	0.59^**^	0.79^**^
7. Aesthetic							1	0.78^**^
8. Free time general								1

^**^p < 0.01 (highly significant).

### Do leisure time satisfaction levels of teachers working in the private sector predict their life satisfaction levels?

The multiple regression analysis demonstrated that the model was statistically significant and was correlated with a substantial proportion of the variance in life satisfaction (*R*^2^ = 0.65, *F*(6, 301) = 93.12, *p* < 0.001). This finding indicates that the psychological, educational, social, relaxation, physical, and aesthetic dimensions collectively were associated with approximately 65% of the variance in life satisfaction. This proportion suggests that the various aspects of leisure time satisfaction are strongly correlated with teachers' overall life satisfaction. The tolerance values ranged from 0.23 to 0.49, while the Variance Inflation Factor (VIF) values ranged from 2.02 to 4.44. Since the VIF values were below 5 and the tolerance values were above 0.20, this indicates that there was no serious multicollinearity issue in the model. Among the dimensions, the psychological (β = 0.35, *p* < 0.001), social (β = 0.31, *p* < 0.001), educational (β = 0.26, *p* < 0.001), and relaxation (β = 0.22, *p* = 0.001) dimensions were all positively and significantly associated with life satisfaction. Conversely, the physical (β = 0.13, *p* = 0.062) and aesthetic (β = 0.09, *p* = 0.153) dimensions did not show significant associations with life satisfaction (see Table 9).

**Table 9:** Multiple regression analysis examining the relationships between dimensions of leisure time satisfaction and overall life satisfaction.

Predictor	B	SE	β	*t*	*p*	Tolerance	VIF
Psychological	0.38	0.07	0.35	5.43	< 0.001	0.23	4.44
Educational	0.29	0.08	0.26	3.63	< 0.001	0.29	3.47
Social	0.33	0.06	0.31	5.50	< 0.001	0.31	3.20
Relaxation	0.24	0.07	0.22	3.43	0.001	0.32	3.12
Physical	0.15	0.08	0.13	1.88	0.062	0.45	2.22
Aesthetic	0.10	0.07	0.09	1.43	0.153	0.49	2.02

*R*^2^ = 0.65, *F*(6, 301) = 93.12, *p* < 0.001.

The analysis revealed that the model was statistically significant. The findings indicated that leisure time satisfaction was associated with approximately 61% of the variance in overall life satisfaction. Thus, higher levels of satisfaction with leisure time were linked to higher levels of life satisfaction among teachers (Table 10).

**Table 10:** Simple linear regression analysis of the relationship between overall leisure satisfaction and life satisfaction.

Predictor variable	B	SE	β	*t*	*p*
Overall leisure satisfaction	0.82	0.04	0.78	21.80	< 0.001

*R* = 0.78, *R*^2^ = 0.61, Adjusted *R*^2^ = 0.61, *F*(1, 306) = 475.41, *p* < 0.001.

## Discussion and conclusion

In this study, we examined the mean scores of teachers on the Leisure Time Satisfaction Scale across various socio-demographic variables. The findings indicated that leisure satisfaction did not differ significantly based on age, marital status, educational background, professional seniority, or duration of employment at the same school. This suggests that these variables are not strongly associated with leisure satisfaction in the current sample.

However, significant differences were observed based on gender. Female teachers reported higher scores in the psychological, educational, relaxation, and aesthetic sub-dimensions, as well as in overall leisure satisfaction. These findings indicate that gender is associated with variations in leisure satisfaction. This pattern aligns with previous studies (Çevik et al., 2021; Sevin and Sahin, 2019). One possible interpretation is that female teachers may engage with or evaluate their leisure experiences differently, particularly regarding emotional recovery and social connectedness. Supporting this idea, Erdemli (2019) described leisure as a psychological support mechanism for teachers, while Öge (2020) highlighted the relationship between leisure satisfaction and cognitive flexibility. Additionally, Abós et al. (2021) demonstrated that participation in structured leisure activities is associated with improved wellbeing outcomes.

No significant gender differences were observed in the social and physical sub-dimensions of leisure satisfaction. This aligns with the findings of Sevin et al. (2020) and Koç and Er (2020), who noted that gender does not consistently impact all aspects of leisure and work-related outcomes. These varied results imply that gender differences in leisure satisfaction may depend on the context and are influenced by various interacting factors, such as organizational roles and workload distribution. Additionally, cultural context may play a role; in Türkiye and the Turkish Republic of Northern Cyprus, for instance, gender roles and societal expectations could affect how leisure is experienced and assessed. However, due to the cross-sectional nature of the study, these interpretations are tentative, and alternative or reciprocal explanations cannot be dismissed.

The findings show that preschool and primary school teachers report higher levels of leisure satisfaction compared to their middle and high school counterparts. This suggests that the school context may be related to differences in leisure satisfaction. Previous research emphasizes the role of institutional and professional factors in shaping leisure experiences (Selçuk and Akdag, 2020; Güvendi, 2023). One possible explanation is that teaching at earlier educational levels often involves more interactive and flexible roles, which could lead to more positive evaluations of leisure time. In contrast, teachers in exam-oriented environments typically face heavier workloads and heightened stress, which may result in lower leisure satisfaction. Supporting this perspective, studies by Zimmermann et al. (2021), Dahl and Karlsudd (2015), and Ackesjö et al. (2019) highlight how institutional structures and professional demands influence teachers' experiences. However, it is essential to approach these interpretations with caution, as the current study design does not allow for causal conclusions.

Work patterns are connected to leisure satisfaction. Full-time teachers reported higher levels of leisure satisfaction in most sub-dimensions and overall scores compared to those working part-time, on contracts, or in other arrangements. This aligns with previous research suggesting that employment conditions affect leisure experiences (Aydin et al., 2019; Kaytan, 2021; Iskender and Güçer, 2018). One possible explanation is that more stable and structured work conditions lead to more favorable evaluations of leisure time. Additionally, full-time employment may provide greater institutional support and foster professional integration. However, it is also possible that individuals with higher wellbeing are more likely to secure or retain full-time positions, or that both leisure satisfaction and employment status are influenced by other factors such as organizational climate or economic conditions.

Significant differences in life satisfaction were observed across various factors. Female teachers, individuals with doctoral degrees, preschool educators, and full-time employees reported higher levels of life satisfaction compared to their counterparts. These findings suggest that life satisfaction is influenced by gender, educational level, type of school, and employment status. Previous studies indicate that factors such as social relationships (Yilmaz and Aslan, 2013) and self-efficacy (Saraçlar et al., 2022) are also related to life satisfaction. One potential explanation is that these factors may coincide with the observed variables and contribute to more positive evaluations of life. However, alternative explanations are also valid. For example, individuals with higher life satisfaction might view their work and leisure experiences more positively. Additionally, unmeasured variables, such as organizational support, income adequacy, or psychological resources, could influence both leisure activities and overall life satisfaction.

The findings of this study indicate that leisure satisfaction and life satisfaction are connected to various individual and institutional factors. While the results do not establish causal relationships, they demonstrate meaningful associations among private school teachers in Northern Cyprus. These findings align with the broader literature while also providing context-specific insights. Future research that employs longitudinal or experimental designs would be beneficial for clarifying the direction and underlying mechanisms of these relationships.

No statistically significant differences were found in life satisfaction scores among different age groups, marital statuses, or levels of professional seniority. This suggests that these demographic factors do not have a strong association with life satisfaction in the current sample. Instead, life satisfaction seems to be more closely connected to individual attitudes, working conditions, and organizational factors. This aligns with findings from previous studies (Gül, 2019; Kurtipek et al., 2023).

In contrast, significant differences were noted concerning educational attainment. Teachers who held doctoral degrees reported higher levels of life satisfaction compared to those categorized in the “other” group. This indicates a positive relationship between higher educational levels and life satisfaction. One possible interpretation is that individuals with advanced education may possess greater cognitive and social resources, leading to more favorable evaluations of their lives. Similar trends have been observed in prior research (Marcionetti and Castelli, 2023; Güvendi, 2023; Selçuk and Akdag, 2020). However, due to the cross-sectional design of the study, alternative or reciprocal explanations cannot be ruled out.

No significant differences were found regarding the length of service at the same school, indicating that continuity of employment is not strongly linked to life satisfaction in this sample. In contrast, notable differences were observed in work patterns, with full-time teachers reporting higher levels of life satisfaction compared to those employed on a contractual or other basis. These findings suggest that the type of employment is associated with variations in life satisfaction, possibly reflecting differences in job security, professional identity, and perceived organizational support. However, because this study used a cross-sectional design, these relationships should be interpreted with caution; alternative explanations—such as reverse or reciprocal influences or the impact of unmeasured variables—are still possible (Kaytan, 2021; Iskender and Güçer, 2018; Firat and Cula, 2016; Sahin and Saridemir, 2017; Karaaslan et al., 2020; Tortumlu and Uzun, 2021; Braun et al., 2020; Dirzyte et al., 2024; Jahan Priyanka et al., 2024). The findings of this study reveal that life satisfaction among teachers is not solely determined by demographic characteristics. Instead, it is more closely linked to individual resources, professional factors, organizational support, and the broader cultural context. Rather than implying causal relationships, these results highlight significant associations between these variables and life satisfaction within the sample. The originality of this study lies in its focus on private school teachers in Northern Cyprus, providing context-specific evidence about how various factors collectively influence life satisfaction. These findings contribute to the existing literature by situating the observed associations within both national and international scholarly discussions, while also acknowledging that alternative or reciprocal relationships could also be possible.

The correlation analysis showed that there is a strong and significant positive relationship between life satisfaction and leisure satisfaction. This result is supported by many past studies (Li et al., 2025; Himmetoglu and Ayhan, 2021; Sabirli et al., 2022; Mutz et al., 2021; Çevik et al., 2021; Sevin and Sahin, 2019; Himmetoglu, 2020; Bae, 2022; Öztürk and Koca, 2019; Newman et al., 2014; Kuykendall et al., 2015; Holder et al., 2009; Iso-Ahola and Mannell, 2004). The strong intercorrelations found among the subdimensions of leisure satisfaction suggest that these aspects are closely related and may represent overlapping elements of a broader concept of leisure satisfaction. This observation aligns with previous research, which indicates that the dimensions of leisure satisfaction are conceptually interconnected and not entirely independent of one another (Beard and Ragheb, 1980). For instance, individuals who derive psychological satisfaction from leisure activities often report higher levels of social, relaxation, or educational satisfaction, as these experiences tend to occur together in leisure contexts.

The originality of this study lies in its demonstration of a strong and statistically significant relationship between leisure satisfaction and life satisfaction among private school teachers in Northern Cyprus. This finding aligns with broader trends observed in existing literature. Specifically, higher levels of life satisfaction are associated with full-time employment, higher educational attainment, and roles at the preschool and primary school levels. These patterns indicate that certain professional and demographic characteristics may be linked to more positive assessments of leisure experiences and overall life satisfaction. However, due to the cross-sectional design of the study, these findings should be regarded as associative rather than causal. Additionally, alternative explanations—such as reverse or reciprocal relationships, or the influence of unmeasured variables—are still possible.

Findings indicate that the psychological, educational, social, relaxation, physical, and aesthetic dimensions of leisure time scale collectively were associated with approximately 65% of the variance in life satisfaction. These findings align with numerous studies (Çetinkaya and Akova, 2020; Yildirim and Latifoglu, 2020; Tokay Argan and Mersin, 2021; Cho, 2020; Serdar et al., 2021; Serdar and Harmandar Demirel, 2022; Newman et al., 2014; Kuykendall et al., 2015; Iskender, 2023; Kavlak et al., 2021; Yetim et al., 2020; Argan et al., 2018; Iwasaki, 2007). The current findings reveal that the psychological, educational, social, and relaxation dimensions are positively linked to life satisfaction. In contrast, the physical and aesthetic dimensions appear to have weaker associations, suggesting that life satisfaction is more closely tied to subjective perceptions, emotional support, and social interactions. These results indicate that the leisure experiences of private school teachers in Northern Cyprus are primarily focused on psychological and social aspects of satisfaction, likely influenced by their professional conditions, workload, and the broader sociocultural context. However, because this study is cross-sectional, these relationships should be viewed as associative rather than causal. It is also possible that teachers with higher life satisfaction perceive their leisure experiences more positively, or that both factors may be influenced by other unmeasured variables.

## Limitations of the study

This study has several limitations that should be considered when interpreting the findings. First, the sample is limited to private school teachers in the Turkish Republic of Northern Cyprus, which means the results cannot be generalized to public school teachers or to other national and institutional contexts. Although stratified sampling was used, participation was voluntary, which may have introduced self-selection bias and impacted the representativeness of the sample. Second, the study relied solely on self-report instruments, including the Leisure Time Satisfaction Scale (LTSS) and the Life Satisfaction Scale (LSS). This reliance may increase the risk of common method variance, as well as social desirability and response biases. Third, the cross-sectional design of the study limits the ability to determine temporal or causal relationships between variables. While a significant association between leisure satisfaction and life satisfaction was found, the direction of this relationship remains unclear. Alternative explanations, such as reverse or reciprocal relationships, are also possible. Another limitation of the study is that additional construct validation procedures, such as confirmatory factor analysis (CFA), were not performed with the current sample. Although the scales used in the study have been validated previously and have shown acceptable psychometric properties in earlier research, their factor structures were not re-examined in this specific participant group. As a result, we cannot confirm whether the dimensional structure of the scales is consistent within this sample. Future studies should conduct CFA and other advanced validity analyses to provide stronger evidence for the construct validity of the measurement tools across different samples and contexts. Forth, the study did not consider potential clustering effects at the school level, which may have influenced the statistical estimates. Additionally, although parametric tests were used, deviations from the assumptions of normality could have affected the robustness of the results. Finally, several relevant variables—such as income level, workload, organizational climate, burnout, and work-life balance—were not included in the model. Including these variables in future studies would provide a more comprehensive understanding of teacher wellbeing.

The findings of this study indicate that leisure time satisfaction is positively associated with teachers' overall life satisfaction. Moreover, several dimensions of leisure satisfaction show significant relationships with life satisfaction. However, these conclusions should be interpreted with caution due to the cross-sectional design of the study, the high intercorrelations among the leisure satisfaction subdimensions, and the lack of additional construct validation analyses within the current sample. Therefore, the results do not support causal interpretations. Despite these limitations, the study adds to the literature by emphasizing the potential importance of leisure experiences for teachers' wellbeing and life satisfaction. Future studies that use longitudinal designs and advanced measurement validation procedures may provide a more comprehensive understanding of these relationships.

## Recommendations

### Recommendations for policymakers and educational administrators

The findings demonstrate a positive correlation between leisure satisfaction and life satisfaction, indicating that enhancing teachers' wellbeing necessitates a comprehensive approach. Schools and policymakers should consider promoting organized leisure opportunities, such as social activities, professional development workshops, and recreational programs, to support teachers' psychological recovery and foster social engagement. Employment conditions are linked to overall wellbeing. Therefore, implementing policies that enhance job security, provide institutional support, and promote work-life balance can lead to greater life satisfaction among teachers. Supporting advanced education opportunities and diversifying in-service training programs can further enhance teachers' professional development and overall wellbeing. Additionally, context-sensitive policies, such as flexible working arrangements and initiatives aimed at achieving a work-family balance, can help address the specific needs of different groups of teachers.

### Recommendations for researchers

Future research should utilize more comprehensive models that incorporate variables such as burnout, organizational commitment, job satisfaction, and psychological wellbeing. This approach will provide a deeper understanding of the complexities surrounding teacher wellbeing. Additionally, exploring mediating and moderating factors (such as social support, emotional intelligence, and motivation) could clarify the relationship between leisure satisfaction and life satisfaction. Comparative studies conducted across different sectors and cultural contexts would also improve the generalizability of the findings.

### Recommendations for future studies

Future research should consider utilizing longitudinal or experimental designs to gain a clearer understanding of the directionality of relationships. Advanced analytical techniques, such as structural equation modeling (SEM), may offer deeper insights into both direct and indirect relationships. Furthermore, qualitative or mixed-method approaches—such as interviews or focus groups—could provide more comprehensive insights into teachers' experiences of leisure and wellbeing.

## Data Availability

The original contributions presented in the study are included in the article/supplementary material, further inquiries can be directed to the corresponding author.
